# Anti-PGL-1 Positivity as a Risk Marker for the Development of Leprosy among Contacts of Leprosy Cases: Systematic Review and Meta-analysis

**DOI:** 10.1371/journal.pntd.0004703

**Published:** 2016-05-18

**Authors:** Maria Lucia F. Penna, Gerson O. Penna, Paula C. Iglesias, Sonia Natal, Laura C. Rodrigues

**Affiliations:** 1 Epidemiology Department, Instituto de Saúde da Comunidade, Universidade Federal Fluminense, Niteroi, Rio de Janeiro, Brasil; 2 Universidade de Brasília, Brasília, DF, Brasil; 3 Universidade Federal de Santa Catarina, Florianópolis, SC, Brasil; 4 Department of Infectious Disease Epidemiology, London School of Hygiene and Public Health, London, England; University of Tennessee, UNITED STATES

## Abstract

**Background:**

There is no point of care diagnostic test for infection with *M*. *Leprae* or for leprosy, although ELISA anti PGL-1 has been considered and sometimes used as a means to identify infection.

**Methods:**

A systematic review of all cohort studies, which classified healthy leprosy contacts, at entry, according to anti-PGL1 positivity, and had at least one year follow up. The outcome was clinical diagnosis of leprosy by an experienced physician. The meta-analysis used a fixed model to estimated OR for the association of PGL-1 positivity and clinical leprosy. A fixed model also estimated the sensibility of PGL-1 positivity and positive predictive value.

**Results:**

Contacts who were anti PGL-1 positive at baseline were 3 times as likely to develop leprosy; the proportion of cases of leprosy that were PGL-1 positive at baseline varied but was always under 50%.

**Conclusions:**

Although there is a clear and consistent association between positivity to anti PGL-1 and development of leprosy in healthy contacts, selection of contacts for prophylaxis based on anti PGL1 response would miss more than half future leprosy cases. Should chemoprophylaxis of controls be incorporated into leprosy control programmes, PGL1 appears not to be a useful test in the decision of which contacts should receive chemoprophylaxis.

## Author Summary

Contacts of leprosy cases are more likely to be infected and develop leprosy. But not everyone infected with *M*. *Leprae* develops clinical leprosy. into clinical disease. We examined and summarized all the eight studies that evaluated how well PGL-1 predicts which contacts of leprosy will become cases. PGL-1 positive contacts were 3 times more likely to develop leprosy; a variable proportion, but less than 30% of the cases were attributed to PGL-1 and less than 45% of the PGL-1 contacts developed leprosy. PGL1 would not be an appropriate test to decide which contacts of leprosy should receive preventive therapy if this was proposed in leprosy control programmes.

## Introduction

Leprosy remains a neglected disease, in some parts of the world [1], with a high new case detection rate in spite of worldwide control efforts [2]. Most cases are concentrated in remote areas [3,4].

At primary care, leprosy diagnosis is clinical: presence of skin lesion(s) with altered or absent sensibility.

Early detection and treatment would reduce transmission. More recently, the idea of leprosy prophylaxis is being promoted as a way of reducing transmission. As in the case of many other neglected diseases, new tools are needed for early detection of cases, if we are to achieve a marked reduction in incidence in a short timeframe. An accurate point of care test for the diagnosis of leprosy disease or infection could have a major impact in detection. Point of care diagnostic tests target biomarkers of infection or disease.

In the early 1980’s, Brett et al. described an ELISA test to detect IgM and IgG antibodies against the phenolic glycolipid (PGL) component of *Mycobacterium leprae*. Earlier serological tests for *M*. *leprae* antigens had shown low specificity and the discovery of PGL test created a substantial expectation, given the high specificity reported initially [5]

In 1998 a dipstick assay was developed to detected anti-PGL-1 [6], as a convenient point of care test. The expectation at the time was that a positive anti-PGL-1 result would indicate infection, and a negative result absence of infection. Recent publications still offer this interpretation [7,8,9].

However, evidence produced since does not confirm such a straightforward interpretation, with variations reported in the validity of the test as a predictor of who will develop leprosy. In this paper, we present results of a systematic review and meta-analysis of the risk of developing leprosy, in leprosy contacts according to anti-PGL-1 test results. This could inform any decision of incorporating or not the dipstick assay for IgM anti-PGL1 in leprosy control programmes.

## Methods

A systematic literature review protocol strategy was developed based on the ‘Preferred Reporting Items for Systematic reviews and Meta-Analyses’ (PRISMA) checklist. The protocol was published in Prospero International prospective register of systematic reviews before its implementation (PROSPERO 2013:CDRD42013005285). We aimed to include all cohort studies, which classified, at entry, healthy leprosy contacts according to anti-PGL1 positivity and had at least one year follow up. The outcome was clinical diagnosis of leprosy by an experienced physician. Studies with no leprosy cases in one of the groups, and those using any antigen other than PGL1 conjugated with bovine serum albumin (BSA) met the exclusion criteria. When more than one paper described the same cohort, we included the one with most information.

We searched PUBMED, EMBASE, LILACS, IMSEAR, WPRIM, WHOLIS, IMEMR and INDMED from 1983, when the technique for detection of anti-PGL-1 was published, to April 2015. The electronic search strategy on PUBMED was:

(("Contact"[Journal] OR "contact"[All Fields] OR "Contact"[Journal] OR "contact"[All Fields]) OR contacts[All Fields]) AND (("leprosy"[MeSH Terms] OR "leprosy"[All Fields]) OR ("leprosy"[MeSH Terms] OR "leprosy"[All Fields] OR ("hansen"[All Fields] AND "disease"[All Fields]) OR "hansen disease"[All Fields])) AND (anti-phenolic[All Fields] OR (phenolic[All Fields] AND ("glycolipids"[MeSH Terms] OR "glycolipids"[All Fields] OR "glycolipid"[All Fields])) OR anti-PGL-1[All Fields] OR PGL-1[All Fields] OR ("immunology"[Subheading] OR "immunology"[All Fields] OR "serology"[All Fields] OR "serology"[MeSH Terms] OR "serology"[All Fields] OR "serologic tests"[MeSH Terms] OR ("serologic"[All Fields] AND "tests"[All Fields]) OR "serologic tests"[All Fields]))

We decided to include papers written in English, French, Spanish or Portuguese. Endnote files kept all selected references and abstracts. Two authors (SN and PI) read the abstracts and selected the papers for inclusion in the review. When they disagreed, a third author (MLFP) reviewed it based on the paper’s full text. These three authors assessed the paper’s full text defining those to include in the systematic review.

One of the authors (MLFP) abstracted the data and another (SN) checked it. Our main measure of association was the odds ratio (OR) and its log transformation (LOR) based on the number of patients at the beginning of follow up in each category (anti_PGL1 positives and negatives) and the number of cases in each category.

We used the Tool to Assess Risk of Bias in  Cohort Studies from Cochrane Bias Methods Group to classify each paper. We did not apply items 4 and 5 since these items were about the presence and control of other prognostic factors, which was not relevant for this review. We also abstracted data about the site of the study, proposed time of follow up, type of antigen used, technique of the test, dilution used and cut off point.

We estimated the summary LOR as the combined inverse-variance weighted LOR of the individual studies, i.e., used a fixed effect model.

As a measure of heterogeneity, we used Cochran’s Q (this has the same distribution as chi square with n -1 degrees of freedom, where n is the number of studies). The set of studies was considered heterogeneous if p<0.1. The inconsistency index was estimated (I^2^) and if the index was 40% or less, we considered that the inconsistency was not important. A funnel plot evaluated publication bias Sensitivity analysis was based on the variation of the summary OR when one study was removed.

We present a ROC plane plot with the results of each study. Ulrich et al. was excluded from the plot because in this study included all contacts with negative reactions to *M leprae* and only a sample of those with positive reaction. The study sample is not balanced in respect of all possible immunological response among contacts, although it has internal validity.

## Results

**Fig 1:**
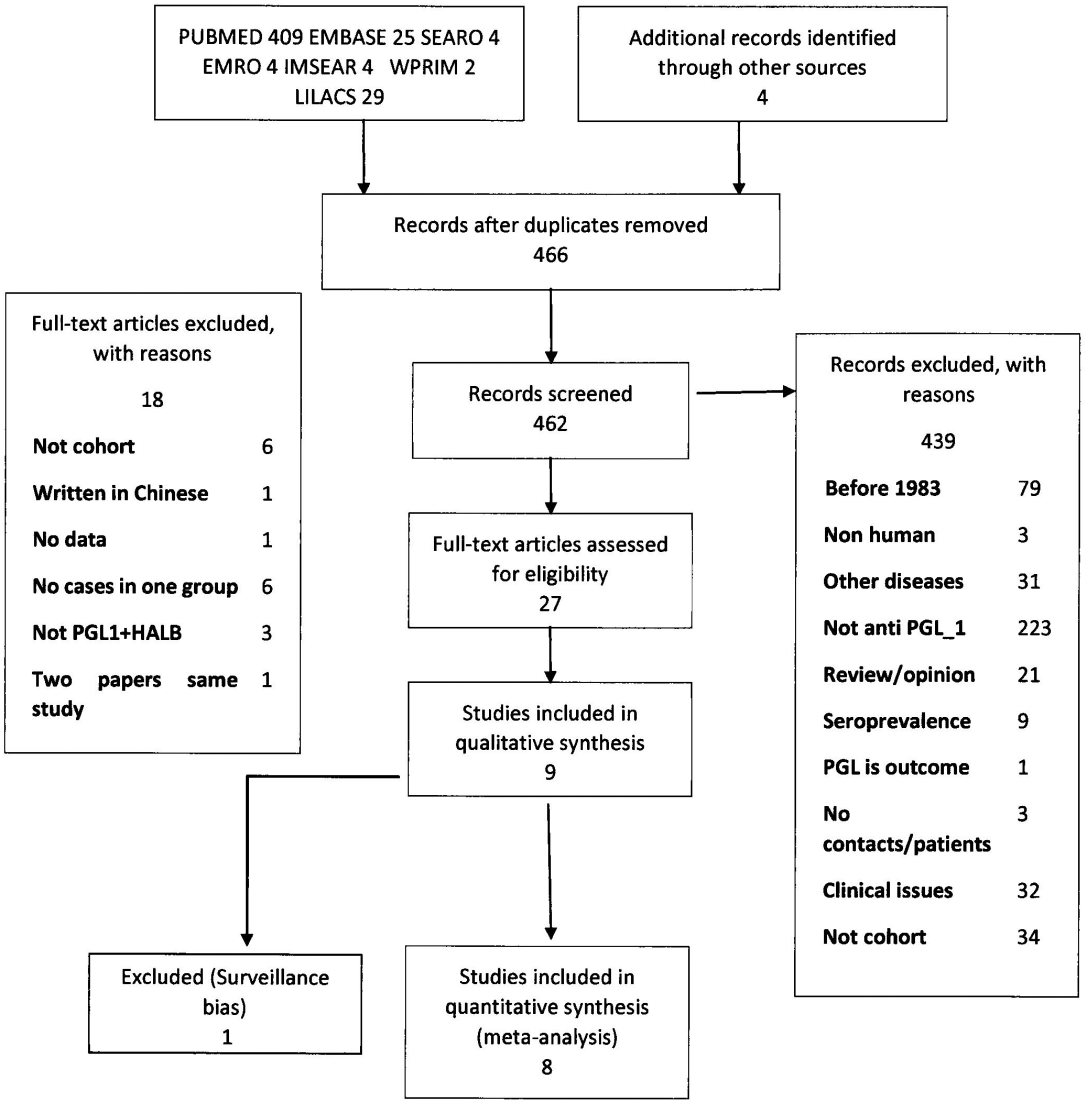
Studies selection flow diagram.

We retrieved abstracts of 462 papers and we selected 27 for full-text reading. From those, 9 were selected for the systematic review and 8 entered for the meta-analysis (Fig 1). We accepted the authors’ definition of household contacts and considered neighbourhood contact if the study selected their sample due to the presence of leprosy cases in an area.

Table 1 presents some characteristics of the selected the studies [10,11,12,13,14,15,16,17,18]. Table 2 shows the extracted data for each study.

**Table 1:** Characteristics of the studies selected in the systematic review.

FIRST AUTHOR	YEAR	PLACE	ANTIGEN	ASSAY	DILUITION	CUT POINT	TIME* (YEAR)	TYPE OF CONTACT	PREVALENCE PGL1 + (%)
**CHANTEAU**	1993	FRENCH POLINESIA	NTP-BSA	ELISA	1:250	0.2	9	NEIGHBORHOOD	20.46
**GRONEN**	1990	YAL, ZAIRE	PGL1-BSA	ELIZA	Missing	0.2	4	NEIGHBORHOOD	6.54
**BAGSHAWE**	1990	KALO, PAPUA NEW GUINEAN	PGL1-BSA	ELIZA	1:100	0.2	2	NEIGHBORHOOD	17.57
**ULRICH**	1991	VENEZUELA	NATIVE PGL-PBS-BSA	ELISA	1:300	0.25	4	HOUSEHOLD	50.34
**BRASIL**^&^	2003	ESTADO SÃO PAULO, BRAZIL	KIT	ULTRAMICRO ELISA	Missing	0.3	4	HOUSEHOLD	10.51
**SINHA**	2004	INDIA	PGL1-O-BSA	ELISA	1:300	0.2	1	NEIGHBORHOOD	1.92
**DOUGLAS**	2004	CEBU, PHILLIPINES	PGL1-O-BSA	ELISA	Missing	0.15	7	HOUSEHOLD	7.71
**GOULART**	2008	UBERLANDIA, BRAZIL	KIT	ML FLOW	-	-	5	HOUSEHOLD	12.31
**DUPPRE**	2012	RIO DE JANEIRO, BRASIL	KIT	ML FLOW	-	-	22	HOUSEHOLD	19.07

* maximum duration of follow up proposed by the authors

^&^ excluded from the final analysis

**Table 2:** Data extracted from the selected papers.

*Study name*	*anti-PGL1+*			*anti-PGL1-*		
	*DISEASE*	*Total*	*%*	*DISEASE*	*Total*	*%*
**CHANTEAU 1993 [**13**]**	4	204	1.96	10	997	1.00
**GROENEN 1990 [**11**]**	1	82	1.22	10	1253	0.80
**BAGSHAWE 1990 [**10**]**	2	97	2.06	12	552	2.17
**ULRICH 1991 [**12**]**	14	3196	0.44	6	6349	0.09
**BRASIL 2003 [**14**]**	10	60	16.67	11	571	1.93
**DOUGLAS 2004 [**15**]**	7	40	17.50	20	519	3.85
**SINHA 2004 [**16**]**	1	26	3.85	58	1351	4.29
**GOULART 2008 [**17**]**	11	153	7.19	17	1243	1.37
**DUPPRE 2012 [**18**]**	19	342	5.56	41	1793	2.29

Table 3 shows the bias assessment of the papers. Brasil et al. paper [14] was excluded from the meta-analysis because the follow up procedures were not the same in those who were PGL-1 positive and those who were negative: the anti-PGL-1 positive group had annual medical consultation scheduled during the four years follow up period, but the anti-PGL-1 negative group received the test result with information about leprosy signs and symptoms, and the PGL-1 results and interpretation, but there was no active follow up and leprosy diagnosis in this group depended on the individual demand for medical consultation. We considered this to be differential follow-up as the leprosy diagnosis strategy introduced severe ascertainment bias and thus excluded the study from the meta-analysis. We considered this differential follow-up. Leprosy diagnosis strategy introduced severe ascertainment bias and excluded the study.

**Table 3:** Risk of bias assement.

*FIRST AUTHOR*	*YEAR*	*Q 1*	*Q 2*	*Q 3*	*Q 6*	*Q 7*	*Q 8*	*PRESENCE OF BIAS*	*OBS*
*CHANTEAU*	1993	YES	YES	YES	YES	PROB YES	YES	NO	
*GRONEN*	1990	YES	YES	YES	YES	PROB YES	YES	NO	Included data: 1984 cohort
*BAGSHAWE*	1990	YES	YES	YES	NO	PROB YES		PROB NO	
*ULRICH*	1991	YES	YES	YES		PROB YES	PROB NO	PROB NO	Vaccine trial. Included data: serology method two.
*BRASIL*	2003	YES	YES	YES	NO	NO	PROB NO	YES	Ascertainment bias
*SINHA*	2004	YES	YES	YES	YES	PROB YES	YES	NO	
*DOUGLAS*	2004	YES	YES	YES	YES	PROB YES	YES	NO	
*GOULART*	2008	YES	YES	NO	YES	PROB YES	YES	PROB YES	2 prevalent cases included
*DUPPRE*	2012	YES	YES	YES	PROB YES	PROB YES	YES	PROB NO	

Q1. Was selection of exposed and non‐exposed cohorts drawn from the same population?

Q 2. Can we be confident in the assessment of exposure?

Q 3. Can we be confident that the outcome of interest was not present at start of study?

Q 6. Can we be confident in the assessment of outcome?

Q 7. Was the follow up of cohorts adequate?

Q 8. Were co‐Interventions similar between groups?

Excluded questions:

Q4. Did the study match exposed and unexposed for all variables that are associated with the outcome of interest or did the statistical analysis adjust for these prognostic variables?

Q5. Can we be confident in the assessment of the presence or absence of prognostic factors?

**Fig 2:**
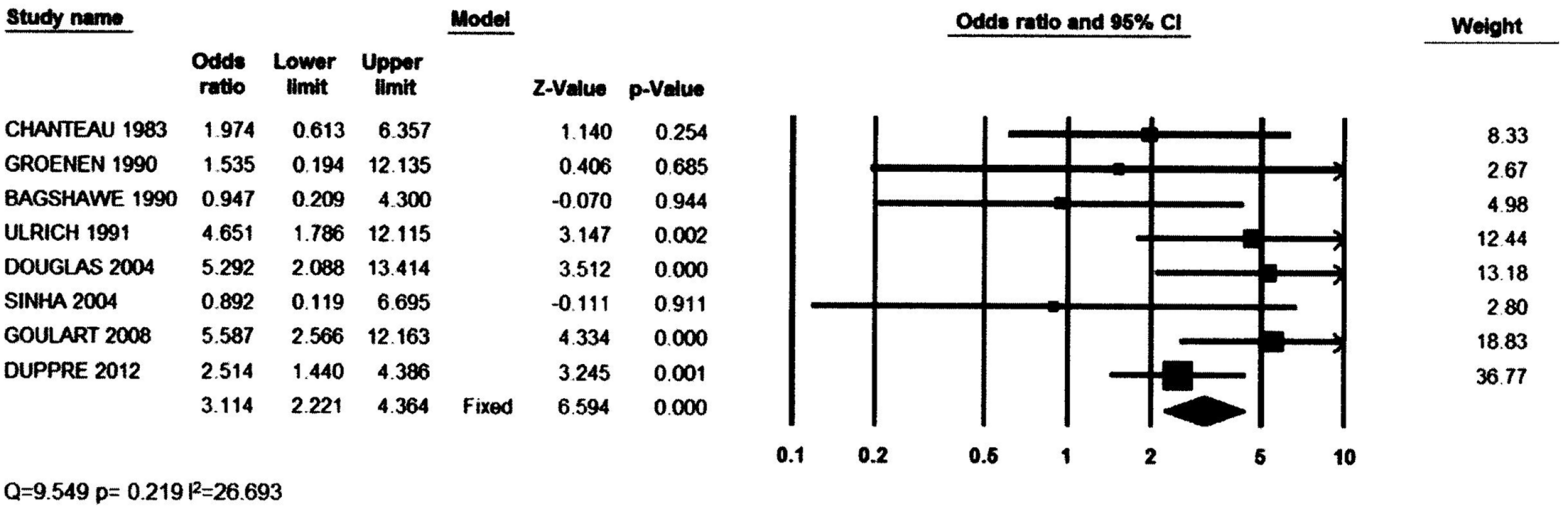
Results of studies and summary OR for leprosy.

**Fig 3:**
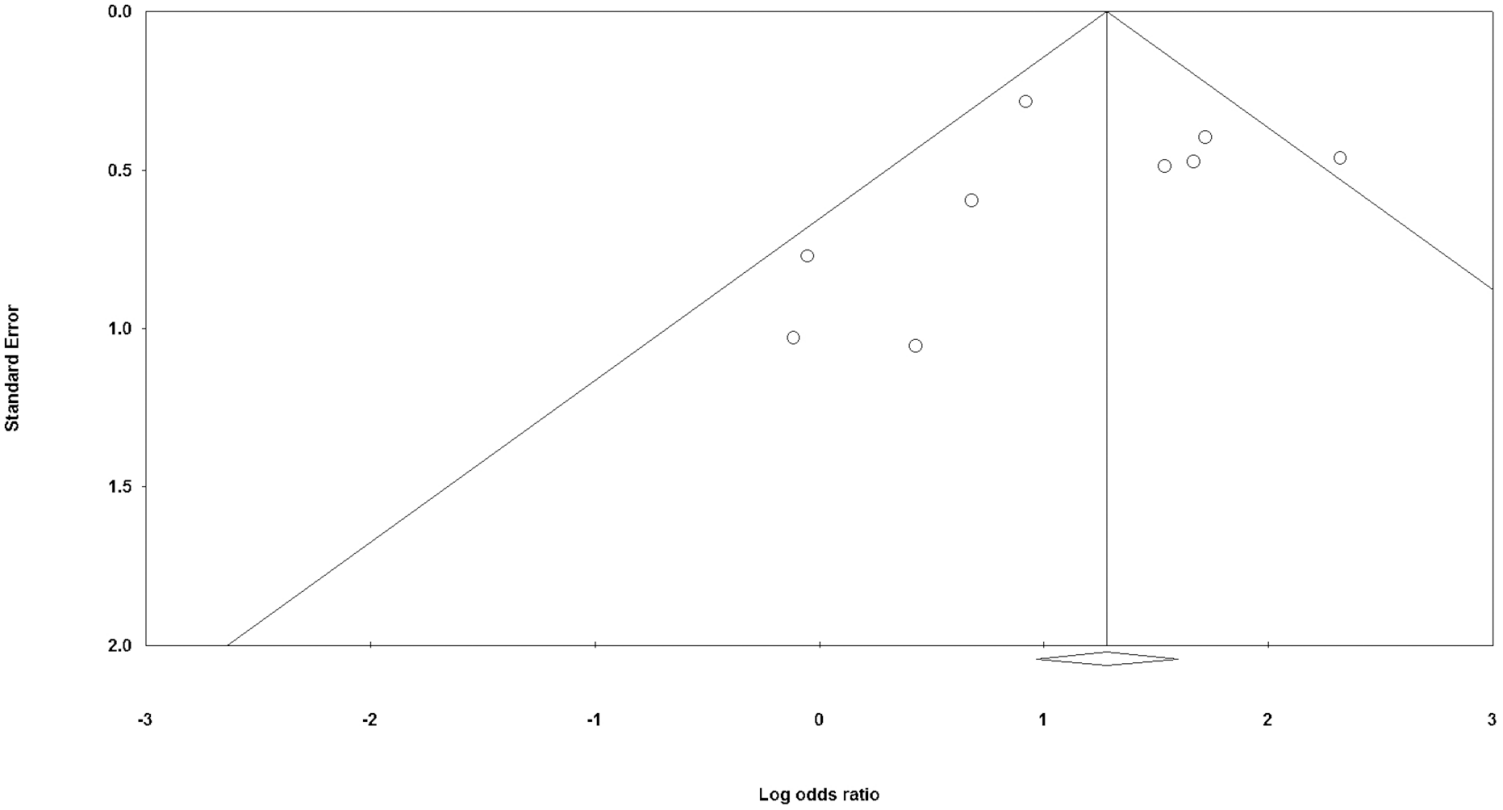
Funnel Plot of each study OR estimate.

Fig 2 shows the forest plot of the included studies. The total number of contacts included in these studies was 18197, with 4140 anti PGL1 positives and 14057 anti PGL1 negatives. The summary ORs with Brasil et al. (14) study removed varied from 2.72 to 3.53, but all the 95% confidence interval included 3.11, the fixed model point estimate. The summary measure with random effects estimate was 3.05 CI95% [1.99–4.67]. The point out of the confidence limit of the funnel plot (Fig 3) represents the excluded paper that had an OR of 10.18.

**Fig 4:**
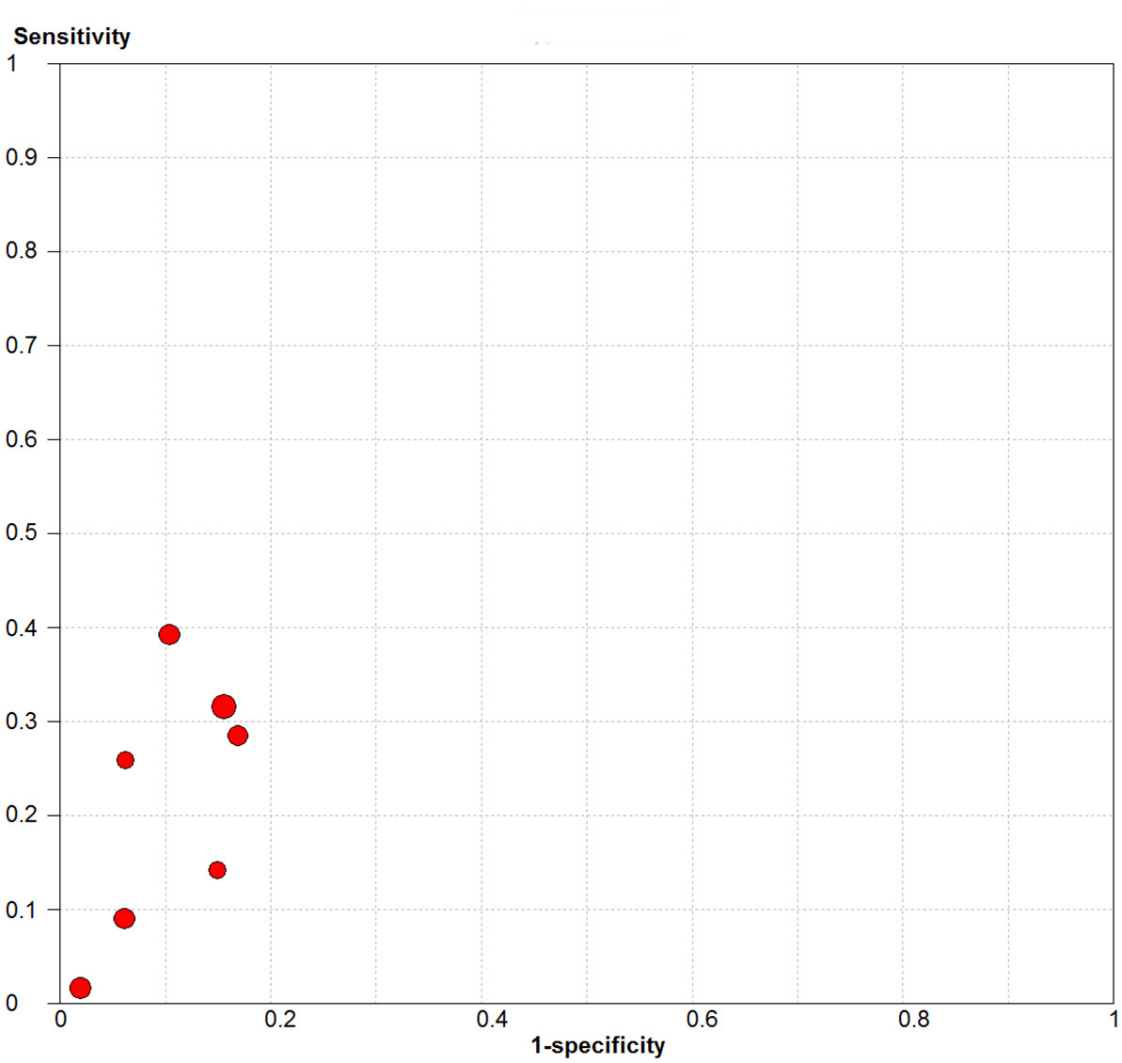
Plot of each study sensitivity and 1-especificity on the ROC Plane.

Fig 4 graphically represents the sensitivity and 1-specificity of each study. The sensitivity varied from 2% [16] to 39% [17] and the specificity from 83% [13]to 98% [16]. Table 4 presents these values and the positive predictive value (PPV) of each study, i.e., the proportion of positives results that developed clinical leprosy. Douglas 2004 is the study with higher PPV due to a high specificity and a moderate sensibility. Chanteau 1993 and Sinha 2004 had higher specificity but a very low sensibility.

**Table 4:** Sensitivity, Specificity and Positive Predictive Valor for each study.

STUDY	Sensitivity	Specificity	PPV
**CHANTEAU 1993 [**13**]**	28.57	82.81	1.96
**GROENEN 1990 [**11**]**	9.09	93.81	1.22
**BAGSHAWE 1990 [**10**]**	14.29	84.72	2.06
**DOUGLAS 2004 [**15**]**	25.93	92.48	17.5
**SINHA 2004 [**16**]**	1.69	98.03	3.85
**GOULART 2008 [**17**]**	39.29	88.82	7.19
**DUPPRE 2012 [**18**]**	31.67	83.52	5.56

## Discussion

The meta-analysis shows that, among healthy contacts of leprosy cases, the risk of developing leprosy is roughly 3 times higher in those who are positive to anti PGL1 than in those who are negative. This was very homogenous. The sensibility of the PGL1 test as a predictor of clinical leprosy development was below 50% for all studies and its specificity was above 80%.

The main methodological limitation of the studies included in this review is the high percentage of losses to follow up in the individual studies. The probability that these losses were associated to the serological result is low, and therefore we consider it unlikely that this would have introduced selection bias. Another limitation is that most papers did not report person years of follow up, so that only the OR could be used as the measure of the association. If we ignore these losses, the summary relative risk would be 3.02 CI 95% [2.2–4.2] (S1 Fig), very close to our summary OR estimate. Given the rarity of leprosy in contacts, we are confident that the OR is a good estimation of the relative risk. Here, the OR is the odds of a positive test among those that will developed clinical relatively to those who will not. We also accept that the risk of developing leprosy changes with time since exposure, which would make analysis of person years by duration of follow up more precise; but in the absence of this necessary information, we suggest that the assumption that the association is constant in time since the first exposure is robust, since duration of follow up was the same for positive and negative controls in all studies. The studies LOR have a very weak correlation with follow up duration (correlation coefficient = -0.0786).

Heterogeneity of the ORs between the studies was not high, with an I^2^ = 26.7, even if different techniques were used for serology, including different antigens. No paper had a clear definition of household or neighbourhood contact—different criteria that would lead to potentially very different level of exposure to leprosy infection in each study. This difference does not bias the estimate of the OR, but is probably the reason for the variation in incidence of leprosy and of the proportion of anti PGL1 positivity between the studies. The sensitivity analysis produced estimates that are included in the 95% CI of the main summary estimates, pointing that the result of the main analysis is not very sensitive to the choice of studies.

Although the OR summarizes the accuracy of the test, as it is here the ratio of sensitivity and one minus specificity, it is difficult to be used for programme decisions. We could not summarize the sensitivity and specificity of all studies because of great heterogeneity (I^2^ = 80,8% for sensitivity and 98% for 1-specificity). This is expected because of the variation of techniques, dilution and cut points used. However, our revision points that we cannot expect a sensitivity over 50%. The reduction of heterogeneity of OR occurs for sensitivity and specificity are correlated.

Routine leprosy control programmes include contact tracing and activities for early diagnosis and prevention of leprosy cases. In addition to clinical examination of contacts for leprosy diagnosis and health education, a few countries include BCG vaccination or revaccination as a prophylactic measure [19]. Another measure often discussed but not yet approved or adopted is chemoprophylaxis with a single dose of rifampicin to treat infection before it develops into leprosy [20]. Currently, the scientific community awaits the result of ongoing controlled trial testing BCG and chemoprophylaxis in the reduction of leprosy in contacts [21]. A collaborative group that includes Novartis Foundation is now implementing a “Leprosy Post-Exposure-Prophylaxis (LPEP)” project that includes pilot subprojects around the world to introduce LPEP in contacts of newly diagnosed cases. The possibility of selecting contacts at higher risk of leprosy for prophylaxis through anti-PGL 1 testing is included in the report of Novartis Foundation Expert Group [22]. We did not find any data supporting this practice, although some authors suggest it [14]. Our findings do not support this recommendation.

Our results show that among the studies included in this review the highest sensitivity is less than 40%. This suggests that selecting contacts positive for anti-PGL for prophylactic measures, would only prevent less than half of leprosy cases among contacts, assuming that the efficacy of chemoprophylaxis in preventing leprosy is 100% This selection would also give chemoprophylaxis unnecessarily to more than 80% (see positive predictive value).

There is an association between anti-PGL 1 positivity and development of leprosy, but we cannot state that anti-PGL 1 result reflects recent infection by *M*.*leprae*. The relationship is more complex and involves host immunity: patient with tuberculoid form (TT) of leprosy are not positive to PGL-1, and for sure, they are infected with *M*.*leprae*. Because of this, the test cannot be used to measure infection rate in communities, as suggested by some papers [9,23]. The fact that the immunological response can vary among leprosy cases allows a hypothesis that anti PGL 1 antibodies production is present when the immunological response of an infected individual is in the lepromatous end of the disease spectrum(LL). If this is the case, why is the proportion of positives contacts that develop the disease not higher? (Table 3).

A large number of serological test for tuberculosis diagnosis were developed and commercialized in many countries, with many claiming high accuracy, but the current evidence do not support these claims [24]. For leprosy there is only a few commercial tests: *Leprosy Detect ELISA Kit* from InBios, USA proposed for diagnosis [25] or screening [26] and *OL Hanseniase* from OrangeLife, Brazil. Is it possible that these are tests in search of an application?

We found few papers analysing the mechanisms and functions of humoral immunity in the interaction of *M*. *leprae* and humans. Studies had shown that antibodies produced by tuberculosis infection/disease target about 0.5% of M. tuberculosis proteome and that the target antigen varies a lot among individuals [27]. Different *M*. *tuberculosis* lineages also produce different immunological response. Antibody-mediated immunity is often found to be irrelevant in the control of the infection of intracellular microorganisms, but the current literature points otherwise [28].

The antibody response in tuberculosis correlates positively with bacillary burden the same way anti PGL1 antibodies in leprosy patients do. This correlation could indicate that healthy contacts positive for anti-PGL1 have been exposed to *M*. *leprae* and have a high bacillary burden. This hypothesis is consistent with the fact that the test detects IgM antibody, an early response to infection and it is therefore interpreted as indicating recent infection. Fig 4 graphically represents the sensitivity and 1-specificity of each study. nevertheless, animal models had shown that IgM antibody might last and participate in long-lasting protection against obligate intracellular bacterium [28]. The hypothesis that not all infected individuals produce anti PGL1 IgM antibodies and that presence can result from both recent and old infection with *M leprae* is plausible and consistent with the lack of ability of anti PGL1 to predict accurately who will and who will not develop leprosy.

### Conclusion

Although there is a clear and consistent increase in risk of development of leprosy in anti PGL-1 positive healthy contacts, selection of cases for prophylaxis intervention based on anti PGL1 response would reach less than half of future leprosy cases, and result in much unnecessary treatment. Leprosy research must explore the role of antibody production in leprosy and it is similar to that in tuberculosis.

## Supporting Information

S1 Fig Meta-analysis using relative risk as association measure. Results and forest plot.
(PDF)

S1 Checklist PRISMA checklist. (PDF)

## References

[1] Penna ML, Penna GO (2012) Leprosy frequency in the world, 1999–2010. Mem Inst Oswaldo Cruz 107 Suppl 1: 3–12. 23283446 10.1590/s0074-02762012000900002

[2] Rodrigues LC, Lockwood DNJ (2011) Leprosy now: epidemiology, progress, challenges, and research gaps. The Lancet Infectious Diseases 11: 464–470. 10.1016/S1473-3099(11)70006-8 21616456

[3] Penna ML, Wand-Del-Rey-de-Oliveira ML, Penna G (2009) Spatial distribution of leprosy in the Amazon region of Brazil. Emerg Infect Dis 15: 650–652. 10.3201/eid1504.081378 19331763 PMC2671445

[4] Worth RM (1996) Leprosy in Hawaii; the end of an epidemic. Int J Lepr Other Mycobact Dis 64: 441–447. 9030111

[5] Brett SJ, Draper P, Payne SN, Rees RJ (1983) Serological activity of a characteristic phenolic glycolipid from Mycobacterium leprae in sera from patients with leprosy and tuberculosis. Clin Exp Immunol 52: 271–279. 6407793 PMC1535852

[6] Buhrer SS, Smits HL, Gussenhoven GC, van Ingen CW, Klatser PR (1998) A simple dipstick assay for the detection of antibodies to phenolic glycolipid-I of Mycobacterium leprae. Am J Trop Med Hyg 58: 133–136. 9502593 10.4269/ajtmh.1998.58.133

[7] Carvalho AP, da Conceicao Oliveira Coelho Fabri A, Correa Oliveira R, Lana FC (2015) Factors associated with anti-phenolic glycolipid-I seropositivity among the household contacts of leprosy cases. BMC infectious diseases 15: 219 10.1186/s12879-015-0955-3 26024906 PMC4449587

[8] Fabri Ada C, Carvalho AP, Araujo S, Goulart LR, de Mattos AM, et al (2015) Antigen-specific assessment of the immunological status of various groups in a leprosy endemic region. BMC infectious diseases 15: 218 10.1186/s12879-015-0962-4 26021317 PMC4448205

[9] Barreto JG, Bisanzio D, Guimaraes Lde S, Spencer JS, Vazquez-Prokopec GM, et al (2014) Spatial analysis spotlighting early childhood leprosy transmission in a hyperendemic municipality of the Brazilian Amazon region. PLoS Negl Trop Dis 8: e2665 10.1371/journal.pntd.0002665 24516679 PMC3916250

[10] Bagshawe AF, Garsia RJ, Baumgart K, Astbury L (1990) IgM serum antibodies to phenolic glycolipid-I and clinical leprosy: two years' observation in a community with hyperendemic leprosy. Int J Lepr Other Mycobact Dis 58: 25–30. 2181044

[11] Groenen G PS, Ghys P et al (1990) A longitudinal study of the incidence of leprosy in a hyperendemic area in zaire. Int J Lepr Other Mycobact Dis 58: 641 2280114

[12] Ulrich M, Smith PG, Sampson C, Zuniga M, Centeno M, et al (1991) IgM antibodies to native phenolic glycolipid-I in contacts of leprosy patients in Venezuela: epidemiological observations and a prospective study of the risk of leprosy. Int J Lepr Other Mycobact Dis 59: 405–415. 1890364

[13] Chanteau S, Glaziou P, Plichart C, Luquiaud P, Plichart R, et al (1993) Low predictive value of PGL-I serology for the early diagnosis of leprosy in family contacts: results of a 10-year prospective field study in French Polynesia. Int J Lepr Other Mycobact Dis 61: 533–541. 8151183

[14] MT L.R.F. (2003) Sorologia Anti PGL-1 e risco de ocorrência de hanseníase em área de alta endemicidade do Estado de São Paulo: quatro anos de seguimento. Revista Brasileira de Epidemiologia 6: 262.

[15] Douglas JT, Cellona RV, Fajardo TT Jr., Abalos RM, Balagon MV, et al (2004) Prospective study of serological conversion as a risk factor for development of leprosy among household contacts. Clin Diagn Lab Immunol 11: 897–900. 15358649 10.1128/CDLI.11.5.897-900.2004 PMC515277

[16] Sinha S, Kannan S, Nagaraju B, Sengupta U, Gupte MD (2004) Utility of serodiagnostic tests for leprosy: a study in an endemic population in South India. Lepr Rev 75: 266–273. 15508903

[17] Goulart IM, Bernardes Souza DO, Marques CR, Pimenta VL, Goncalves MA, et al (2008) Risk and protective factors for leprosy development determined by epidemiological surveillance of household contacts. Clin Vaccine Immunol 15: 101–105. 17989339 10.1128/CVI.00372-07 PMC2223848

[18] Duppre NC, Camacho LA, Sales AM, Illarramendi X, Nery JA, et al (2012) Impact of PGL-I seropositivity on the protective effect of BCG vaccination among leprosy contacts: a cohort study. PLoS Negl Trop Dis 6: e1711 10.1371/journal.pntd.0001711 22724040 PMC3378622

[19] Sales AM, Ponce de Leon A, Duppre NC, Hacker MA, Nery JA, et al (2011) Leprosy among patient contacts: a multilevel study of risk factors. PLoS Negl Trop Dis 5: e1013 10.1371/journal.pntd.0001013 21423643 PMC3057944

[20] Moet FJ, Pahan D, Oskam L, Richardus JH, Group CS (2008) Effectiveness of single dose rifampicin in preventing leprosy in close contacts of patients with newly diagnosed leprosy: cluster randomised controlled trial. BMJ 336: 761–764. 10.1136/bmj.39500.885752.BE 18332051 PMC2287265

[21] Richardus RA, Alam K, Pahan D, Feenstra SG, Geluk A, et al (2013) The combined effect of chemoprophylaxis with single dose rifampicin and immunoprophylaxis with BCG to prevent leprosy in contacts of newly diagnosed leprosy cases: a cluster randomized controlled trial (MALTALEP study). BMC Infect Dis 13: 456 10.1186/1471-2334-13-456 24088534 PMC3850918

[22] Smith WC, Aerts A (2014) Role of contact tracing and prevention strategies in the interruption of leprosy transmission. Lepr Rev 85: 2–17. 24974438

[23] Barreto JG, Guimaraes Lde S, Frade MA, Rosa PS, Salgado CG (2012) High rates of undiagnosed leprosy and subclinical infection amongst school children in the Amazon Region. Mem Inst Oswaldo Cruz 107 Suppl 1: 60–67. 23283455 10.1590/s0074-02762012000900011

[24] Steingart KR, Flores LL, Dendukuri N, Schiller I, Laal S, et al (2011) Commercial serological tests for the diagnosis of active pulmonary and extrapulmonary tuberculosis: an updated systematic review and meta-analysis. PLoS Med 8: e1001062 10.1371/journal.pmed.1001062 21857806 PMC3153457

[25] Duthie MS, Raychaudhuri R, Tutterrow YL, Misquith A, Bowman J, et al (2014) A rapid ELISA for the diagnosis of MB leprosy based on complementary detection of antibodies against a novel protein-glycolipid conjugate. Diagn Microbiol Infect Dis 79: 233–239. 10.1016/j.diagmicrobio.2014.02.006 24666703

[26] de Souza MM, Netto EM, Nakatani M, Duthie MS (2014) Utility of recombinant proteins LID-1 and PADL in screening for Mycobacterium leprae infection and leprosy. Transactions of the Royal Society of Tropical Medicine and Hygiene 108: 495–501. 10.1093/trstmh/tru093 24907710

[27] Kunnath-Velayudhan S, Salamon H, Wang HY, Davidow AL, Molina DM, et al (2010) Dynamic antibody responses to the Mycobacterium tuberculosis proteome. Proceedings of the National Academy of Sciences of the United States of America 107: 14703–14708. 10.1073/pnas.1009080107 20668240 PMC2930474

[28] Chan J, Mehta S, Bharrhan S, Chen Y, Achkar JM, et al (2014) The role of B cells and humoral immunity in Mycobacterium tuberculosis infection. Seminars in immunology 26: 588–600. 10.1016/j.smim.2014.10.005 25458990 PMC4314354

