# Supplementary figures and images for: Anti-PGL-1 Positivity as a Risk Marker for the Development of Leprosy among Contacts of Leprosy Cases: Systematic Review and Meta-analysis
Source: PLoS Negl Trop Dis. 2016 May 18;10(5):e0004703. doi: 10.1371/journal.pntd.0004703 (PMC4871561; doi:10.1371/journal.pntd.0004703)

# Assumption: no lost of follow-up

## Meta Analysis

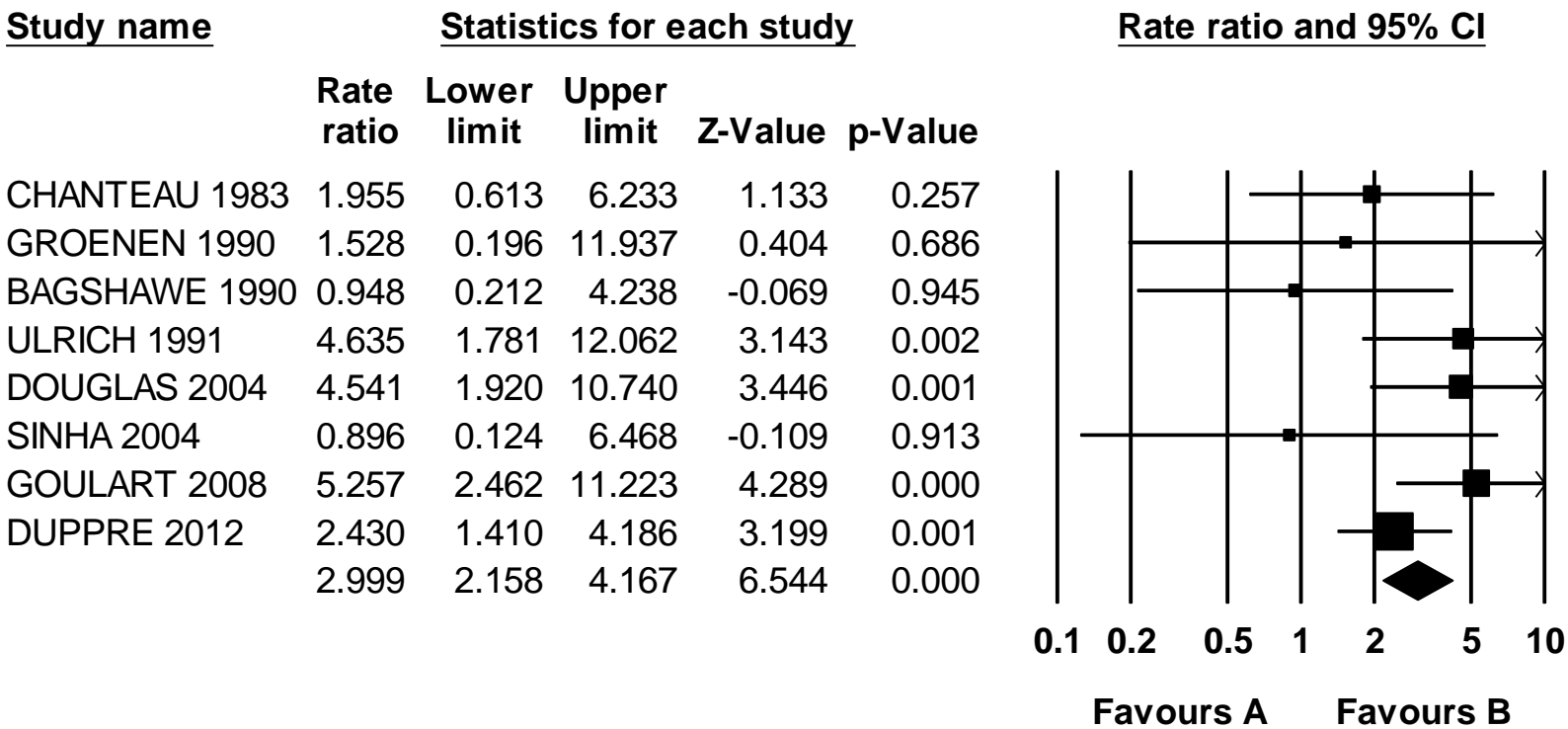

Supplement: S1 Fig — Results and forest plot. (PDF) [file pntd.0004703.s001.pdf]
